# Review and Evaluation of Online Tobacco Dependence Treatment Training Programs for Health Care Practitioners

**DOI:** 10.2196/jmir.3284

**Published:** 2015-04-17

**Authors:** Peter Selby, Karina Goncharenko, Megan Barker, Myra Fahim, Valerie Timothy, Rosa Dragonetti, Katherine Kemper, Marilyn Herie, J Taylor Hays

**Affiliations:** ^1^Centre for Addiction and Mental HealthAddictions DivisionToronto, ONCanada; ^2^Mayo ClinicGlobal BridgesRochester, MNUnited States; ^3^Centennial CollegeCommunity ServicesToronto, ONCanada; ^4^Mayo ClinicNicotine Dependence CenterRochester, MNUnited States

**Keywords:** distance education, tobacco use, health care, smoking cessation, tobacco dependence, program evaluation, continuing medical education

## Abstract

**Background:**

Training health care professionals is associated with increased capacity to deliver evidence-based smoking cessation interventions and increased quit rates among their patients. Online training programs hold promise to provide training but questions remain regarding the quality and usability of available programs.

**Objective:**

The aim was to assess the quality of English-language online courses in tobacco dependence treatment using a validated instrument.

**Methods:**

An environmental scan was conducted using the Google search engine to identify available online tobacco dependence treatment courses. The identified courses were then evaluated using the Peer Review Rubric for Online Learning, which was selected based on its ability to evaluate instructional design. It also has clear and concise criteria descriptions to ensure uniformity of evaluations by trained experts.

**Results:**

A total of 39 courses were identified, of which 24 unique courses were assessed based on their accessibility and functionality during the period of evaluation. Overall, the course ratings indicated that 17 of 24 courses evaluated failed to meet minimal quality standards and none of the courses evaluated could be ranked as superior. However, many excelled in providing effective navigation, course rationale, and content. Many were weak in the use of instructional design elements, such as teaching effectiveness, learning strategies, instructor’s role, and assessment and evaluation. Evaluation results and suggestions for improvement were shared with course administrators.

**Conclusions:**

Based on the courses evaluated in this review, course developers are encouraged to employ best practices in instructional design, such as cohesiveness of material, linearity of design, practice exercises, problem solving, and ongoing evaluation to improve existing courses and in the design of new online learning opportunities.

## Introduction

Tobacco use remains a global preventable cause of disease, disability, and death. It is estimated to kill 6 million people worldwide annually, most in the developing world where the prevalence of smoking remains high [1]. The World Health Organization’s Framework Convention on Tobacco Control (FCTC-Article 14) highlights the need for countries to provide widely accessible cessation services to address the high level of global tobacco use. However, in many countries throughout the world, tobacco dependence treatment services are not well developed and the main pathway of accessing treatment for tobacco dependence is through the clinical interactions between a doctor and patient. There is evidence that training health care professionals in smoking cessation interventions is associated with increased quit rates in smokers [2] and that individuals who smoke are more likely to make an attempt to quit when advised to do so by a health care practitioner [3,4]. Despite the global health and financial burden of disease from tobacco use, the desire of most patients to quit, and routine contact between health care practitioners and patients, few practitioners are appropriately trained or feel confident to effectively treat tobacco addiction in their patients [4]. To address the high rates of tobacco prevalence and increase cessation levels among individuals who smoke, appropriate and effective training for health care practitioners in tobacco dependence treatment is pertinent.

Successful cessation outcomes can be directly correlated to training health care practitioners in evidence-based tobacco dependence treatment. A Cochrane Review [2] of studies measuring the effectiveness of tobacco dependence treatment training on successful cessation outcomes found that in nearly all the studies reviewed, smoking cessation activities of health care practitioners (including psychosocial and pharmacological interventions) increased dramatically posttraining. Moreover, a study by Olano-Espinosa and colleagues [5] found that training primary care health care practitioners in tobacco dependence treatment had a statistically significant effect on sustained abstinence after 6 months (2.1% in trained group vs 0.3% in the comparison group). These studies suggest that training health care practitioners in tobacco dependence treatment has a direct impact on increased levels of cessation activities among practitioners and successful quit attempts among patients. Given that health care practitioners in any clinical setting are well-suited to engage their patients in tobacco dependence treatment, training reinforces the notion to go beyond simply inquiring about tobacco use status and to instead offer evidence-based treatment. Online tobacco intervention training courses, similar to other e-learning programs, provide many potential benefits such as reaching greater numbers of practitioners from different disciplines, reducing the time required for practitioners to dedicate to improving skills and knowledge compared to in-class courses, and providing a cost-effective option to learning (less time away from patients, courses can be started and paused at any time, no travel required, smaller fees compared to a classroom-based course). Further evidence suggests that online continuing education courses, through incorporation of interactive components, are at least as effective as traditional classroom-based courses [6,7]. To increase reach and educate health care providers in effective tobacco interventions, there has been an increase in Internet-based instruction as part of continuing professional development. Online learning or e-learning is characterized by the use of telecommunication technology to deliver information for education and training and has emerged as the exemplar of modern education [8]. Studies have shown that offering health care providers online tobacco dependence intervention training that builds on current knowledge and skills improved the participants’ attitudes and increased self-efficacy in delivering tobacco dependence treatment [9]. Not only did participants report significantly higher positive attitudes and improved self-efficacy for delivering tobacco dependence treatment services posttraining, but they also demonstrated increased delivery of these interventions with clients [9].

A common criticism of online courses is that they have inadequate evaluation mechanisms to enhance the learner’s ability to apply the knowledge gained and instead merely provide facts. This is especially true when training health care practitioners who see themselves as problem solvers and want practical information to address problems commonly encountered in their practices. Unfortunately, there is no accreditation standard or similar quality measure that allows potential end users to know whether a course has been designed using the accepted best practices for online course design and educational theory. A thorough curation of available online tobacco cessation courses ensures more rigorous standards for delivery of tobacco cessation knowledge and best practices to health care providers via an online format. Therefore, as a first step in assessing online course quality, we set out to review and rank all available English-language online courses in tobacco addiction treatment training using a validated instrument. Secondary aims were to provide feedback to course developers about recommendations for course improvement.

## Methods

### Online Tobacco Course Identification (Environmental Scan)

We conducted a basic search on the Google search engine using variations of “online smoking cessation courses for health care practitioners,” “smoking cessation course,” and “smoking cessation online course/training” keywords and followed links to courses and online resources for practitioners and clients related to smoking cessation embedded in government-funded or -supported websites (eg, Ontario Tobacco Research Unit course) or resource sections of other online course websites. The search to identify online courses available to practitioners seeking an e-learning module was conducted in August 2012. An online course description, estimated time to complete, developer information, the language of instruction, availability of continuing medical education (CME) credits, and course fees were included in the analysis (Multimedia Appendix 1).

### Online Tobacco Course Evaluation

Once the courses were identified, the course director or contact person listed for the course was sent an email invitation to participate (Multimedia Appendix 2). We sent 2 emails as reminders. Evaluation of online-based training courses was then carried out by the evaluation team of the Peer Review Rubric for Online Learning (developed by Towson University Faculty in Maryland) [10]. This rubric was chosen based on its ability to evaluate both instructional design and instructional content of an online course, ability to apply the rubric to a variety of online learning programs, and its clear and concise criteria descriptions to ensure uniformity of evaluations. Moreover, the developers trained raters in the use of the instrument thereby reducing the risk of bias by the investigators and authors. The course evaluations addressed the extent to which each online course adhered to best practices in online teaching/learning, including instructional design enhancements such as the presence of case studies, practice exercises, clinical simulations and demonstrations, video demonstration, and group discussion or consultation, as well as content subject matter relating to the science, policy, and practice of tobacco prevention and smoking cessation. Each of the 16 categories evaluated by the rubric (Textbox 1) allowed for a minimal score of 0 (=not included) and a maximal score of 4 (=superior). The overall rating score allowed for a maximum of 64 points: 54-64=superior, 48-53=above average-good, 41-47=average-OK, 35-40=a start, but needs polish, ≤34=redo entirely. The developers of the rubric opted to use a nonstandardized measure to score the courses because the scale was based on the points within the rubric that were assigned to a percentage range. For example, superior points were based on achieving 85% of the maximum of 64 points (range 54-64 points). The reviewers also established a minimal changes score by highlighting categories where minimal changes could provide maximum points. Detailed examples and comments were then provided on possible changes to enhance the course. Developers of online courses who agreed to participate in this evaluation had the option to receive individualized feedback and information about additional resources/contacts if they wished to engage in quality improvement of their online modules. Those interested in discussing the results of the evaluation in more detail were invited to participate in an in-depth discussion via teleconference.

The external course evaluation/rating team had approximately 15 years of experience in the use of the rubric and established 95% reliability based on few differences in rating scores. The course evaluation/rating team was comprised of senior authors for the rubric who are nationally certified as Peer Reviewers and Master Reviewer. Furthermore, the rating team had no previous knowledge of the content of the courses being considered for review and no prior experience working for or with the authors of this paper.

**Categories of evaluation outlined by the Peer Review Rubric for Online Learning.**

1. Navigation

2. Course rationale

3. Learning and teaching theories

4. Instructional design

5. Goals and objectives

6. Learning strategies

7. Content

8. Interactivity

9. Use of mediated resources and Web

10. Assessment and evaluation

11. Internal organization and consistency

12. Responsiveness to learner’s needs

13. Instructor’s role

14. Teaching effectiveness

15. How to get help

16. Esthetics

## Results

**Figure 1:**
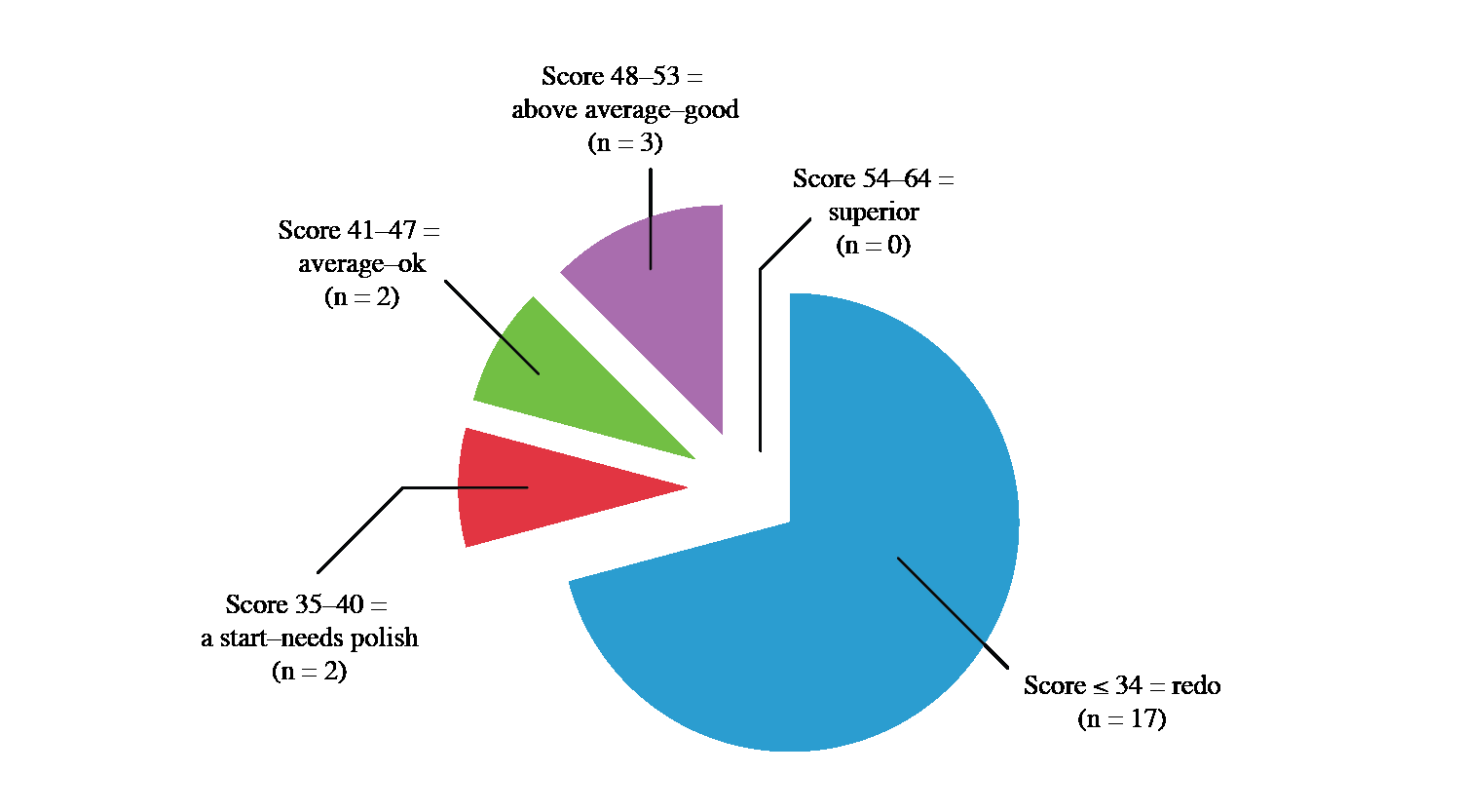
Distribution of courses evaluated by course quality without suggested changes.

We identified 39 courses in English that met our initial criteria. Each of the 39 online courses ranged in length from 1 hour to 20 hours to complete. A total of 27 courses were found to be unique, accessible, and functioning by the end of April 2013. Of these, 3 courses had partial issues of accessibility, such as virus interference and inability to continue to the next module or progress with course material. As a result, we completed evaluations for 24 unique courses (Table 1). Overall course ratings revealed that the majority of courses (17 courses) failed to meet minimal quality standards and needed complete revision, 2 courses were below average (“need polish”), 2 courses were “average-OK,” and only 3 courses ranked as “above average-good” with none being “superior” (Figure 1).

**Table 1:** Course scores as assessed with the Peer Review Rubric for Online Learning.

Course	Evaluation category (score range 0-4)																Course score (max=64)	Minimal changes score (max=64)
	1	2	3	4	5	6	7	8	9	10	11	12	13	14	15	16		
1	3	4	4	4	3	3	4	3	2	2	3	3	4	2	3	4	51	61
2	2	4	4	2	1	3	4	3	3	2	2	3	3	2	3	2	43	51
3	4	2	2	1	0	0	2	2	2	2	0	0	1	0	0	3	21	29
4	4	4	3	1	2	1	3	1	1	2	2	2	1	0	0	2	29	43
5	4	4	3	1	0	1	3	1	1	1	1	2	1	0	0	2	25	40
6	2	3	0	1	0	1	1	0	1	2	1	1	1	0	0	1	15	26
7	3	2	3	3	2	1	3	1	1	2	2	2	1	1	1	3	31	49
8	3	4	3	3	3	2	3	2	2	1	3	3	3	0	3	3	41	48
9	2	3	3	2	1	1	3	1	2	1	3	1	1	1	2	2	29	40
10	2	3	2	1	1	0	2	1	2	1	2	2	2	2	1	1	24	37
11	4	4	4	4	4	2	3	3	3	2	3	3	1	3	4	4	51	56
12	2	4	2	3	1	1	3	1	1	1	3	1	1	1	1	2	28	42
13	3	2	0	2	1	0	1	0	1	1	1	0	1	0	1	1	15	18
14	3	1	3	1	1	1	3	2	2	1	1	2	1	2	0	3	27	40
15	2	4	4	3	1	2	3	2	2	1	1	2	1	1	3	1	33	46
16	4	4	4	4	4	3	4	4	2	2	3	3	2	2	0	3	48	58
17	2	3	1	1	1	0	1	0	0	1	0	0	1	0	2	1	14	25
18	3	4	1	1	0	1	3	1	1	1	3	1	1	0	2	2	25	32
19	3	4	3	2	4	1	3	1	2	3	4	2	1	2	1	2	38	49
20	3	4	3	1	2	2	3	2	2	2	2	2	1	2	4	3	38	47
21	2	4	3	1	2	1	3	2	2	0	1	1	1	0	1	3	27	33
22	3	2	3	2	2	2	2	1	1	0	3	3	1	0	3	2	30	42
23	2	4	0	2	3	1	2	2	1	1	2	0	1	1	2	1	25	47
24	2	4	2	1	3	0	3	2	0	1	1	0	1	2	4	2	28	34

**Figure 2:**
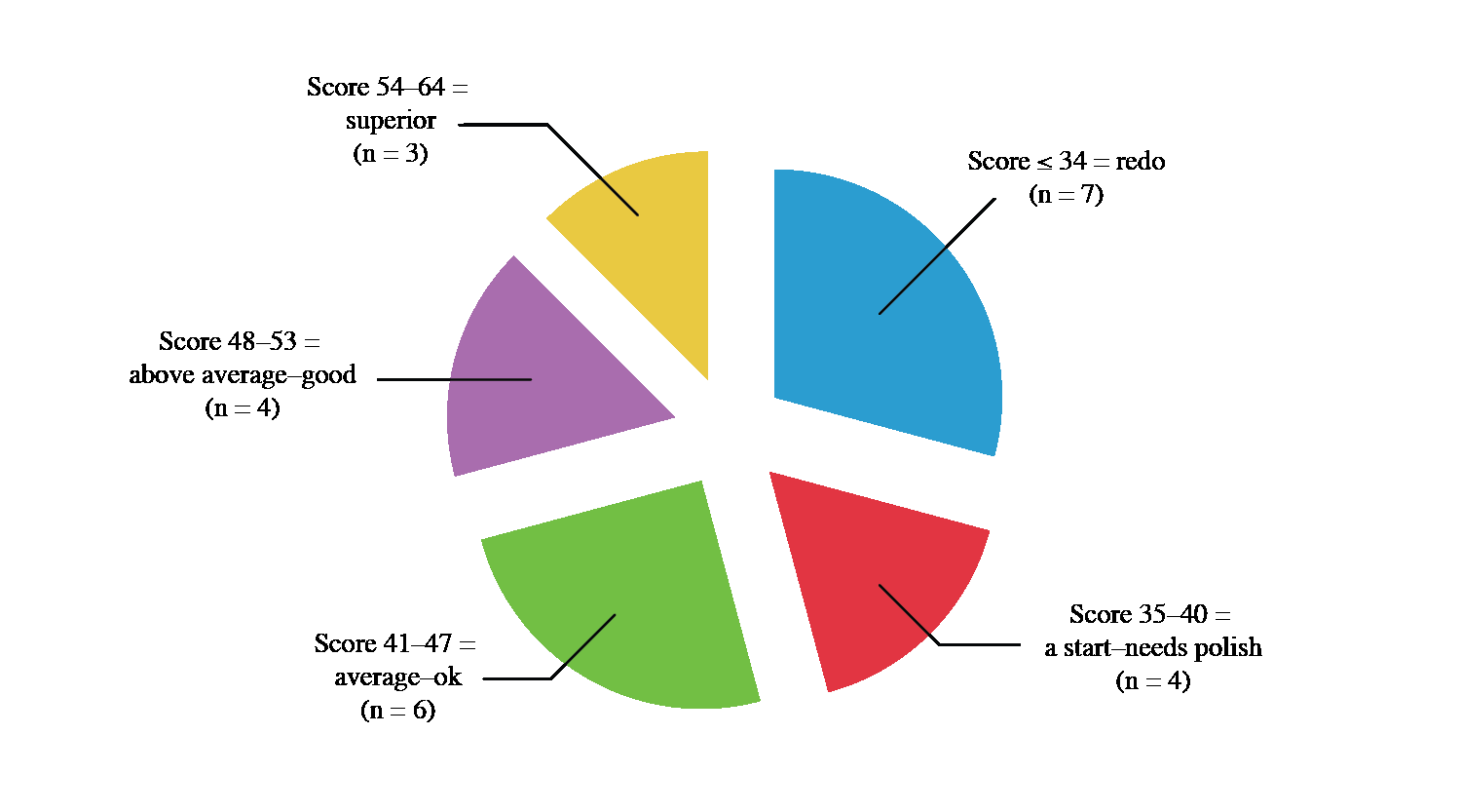
Distribution of courses evaluated by course quality with suggested changes.

To facilitate improvement of the online courses without extensive redevelopment, moderate and quickly implementable amendments were suggested for each of the courses evaluated. These minimal changes were determined at the discretion of the rubric experts. An example of a minimal change suggested by the reviewers was to offer more for learner-led opportunities, such as discussion boards or self-reflective questions to maximize transformational learning. The 3 courses that were rated most highly required only minor changes to become “superior” (Figure 2). Furthermore, with minimal changes, most of the 17 courses with unacceptably low scores could improve their quality rating: 4 courses could be improved to “needs polish,” 6 courses could improve to “average-OK,” and 4 courses could achieve a rating of “above average-good.”

**Figure 3:**
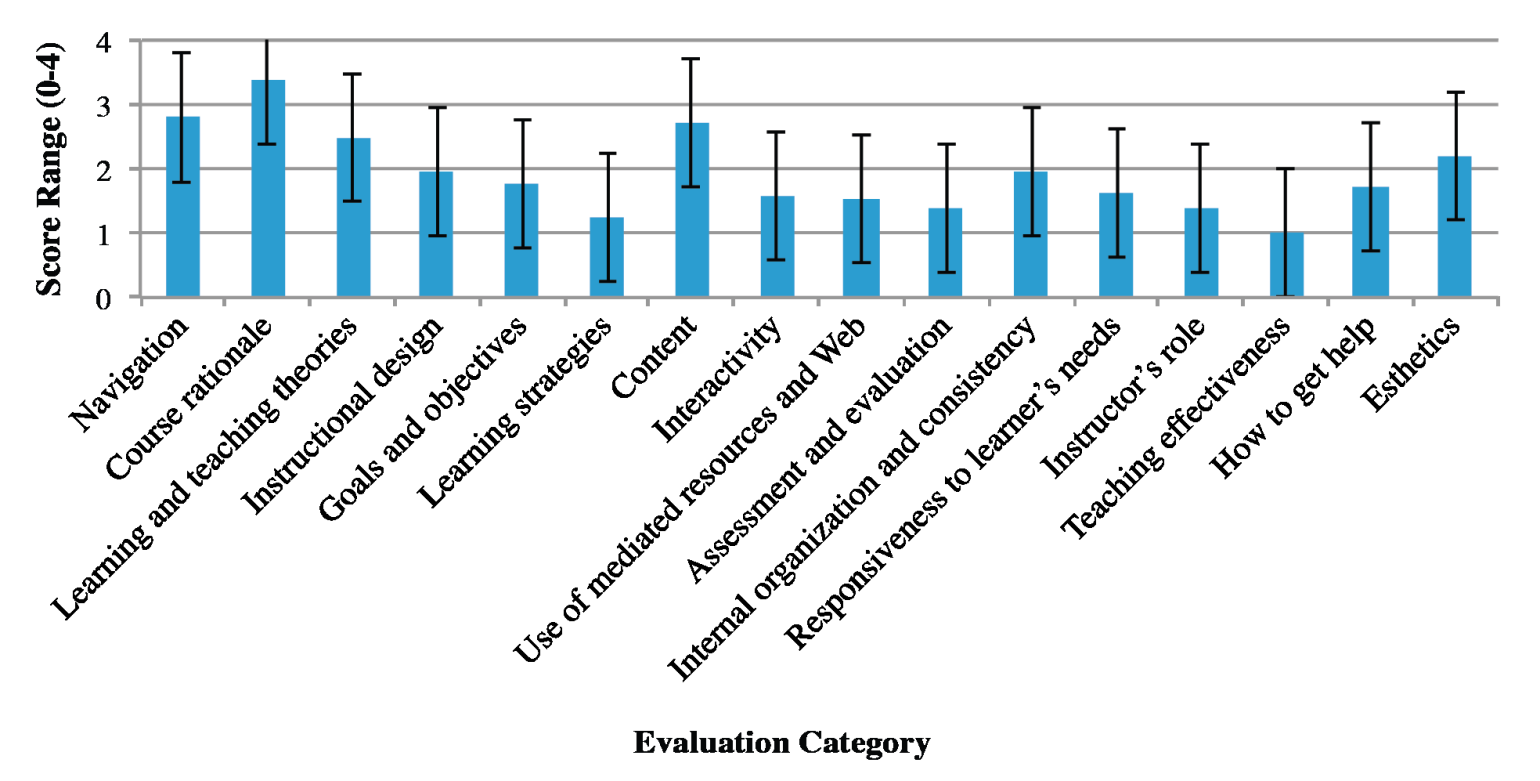
Mean category scores for 24 courses based on the 16 categories evaluated. Error bars indicate standard deviation.

Figure 3 presents mean scores for each category assessed by the rubric. On average, most courses scored well in course rationale, navigation, and content, whereas teaching effectiveness, learning strategies, assessment and evaluation, and instructor’s role proved to be challenging and underdeveloped in most courses. Overall, on the scale of 0-4, most courses scored higher than 2 in 5 categories, scored exactly 2 in 2 categories, and received a score less than 2 on the scale in 9 categories.

Online training programs are often defined by 2 types of e-learning: asynchronous and synchronous. Asynchronous e-learning occurs when students begin and end a training program at different times according to their own schedule and personal preferences. Common features of asynchronous learning include online bulletin and discussion boards, group forums, and self-directed learning. Approximately 92% (22/24) of courses reviewed in this evaluation were categorized as asynchronous. Synchronous e-learning occurs when remote students enroll in a course that follows a specific schedule as outlined by the course instructor. Failure to virtually attend the course or complete assignments according to the scheduled deadlines could result in negative course outcomes. Common features of synchronous learning include live facilitated discussion via whiteboards, virtual classrooms that promote instructor-to-learner and learner-to-learner engagement, and scheduled learner evaluation. Approximately 8% (2/24) of the courses reviewed were categorized as synchronous. Given the number of asynchronous courses reviewed, it is no surprise that 96% (23/24) of the courses evaluated were categorized as self-study. Only 1 course could be categorized as offering collaborative learning because it encouraged learner-to-learner participation in a synchronous virtual classroom in which students were asked to respond to one another’s questions and provide feedback in real time.

Of the 24 courses included in the review, 17 agreed to be evaluated and were provided with individualized feedback and information on additional resources that could improve their course overall. When providing feedback, all courses participating in the review process were offered the opportunity to engage in a more in-depth discussion regarding their evaluation by teleconference. Only 1 of the 17 courses chose the option to further discuss their course review.

## Discussion

Tobacco use and dependence remains the most important cause of preventable death and morbidity worldwide. Despite this fact, many health professionals do not provide effective treatment to tobacco users, in part because of inadequate knowledge and skills in treating the problem. Online education providing instruction in tobacco dependence treatment is one important way to build the necessary capacity to provide evidence-based treatment for tobacco dependence. The online learning environment overcomes many of the barriers found in traditional instruction by expanding the reach and convenience of the instructional material for adult learners. However, adult learners also need to be able to readily access high-quality online learning opportunities to maximize the limited time available for acquiring new knowledge and skills. Unfortunately, the main finding of our review of available online courses for tobacco dependence treatment was not encouraging in this regard. Out of the initial 39 courses identified, a mere 24 courses were able to be accessed for evaluation. The primary obstacle to these courses was inability to access course content due mostly to inadequate course maintenance (ie, ensuring the webpage is virus-free, links are in working condition). Among the 24 courses we evaluated, the majority (17/24) require complete revision to meet minimal quality standards for best practices and optimal instructional design. Fortunately, we also found that there would be a significant positive impact on course quality (an increase of 25% in quality rating scores) with modest revisions. This suggests that there exists a relatively easy way to improve most available courses to meet the average industry standard. A detailed analysis of each of the 16 categories of the assessment rubric makes it clear that most online courses will obtain the greatest benefit by improving in the areas of teaching effectiveness, learning strategies, instructor’s role, and assessment and evaluation. Course developers are encouraged to focus on a wider range of design elements necessary for a successful course (eg, interactivity, aligning learning strategies).

A review of e-learning literature for health care practitioners suggests areas for quality improvement comparable to those found in our study [11]. For example, the review noted that learner testing and assessment is crucial to ensure that information has been absorbed and will be applied, and learners need to receive feedback on their performance. Among the lowest ranking areas found in our study was “assessment and evaluation.” Similarly, low-ranking categories found in our study were instructor’s role, teaching effectiveness, and responsiveness to learner’s needs. The review also found that instructors and developers need to be flexible to adapt to the needs of learners and that learner participation and interaction needs to be supported and encouraged similar to that of traditional classroom environments [11]. Collaboration between content, pedagogy, and technology is needed to keep learners engaged [11]. We found that course interactivity, learning strategies, and instructional design—features that enhance learner engagement—were lacking for most tobacco dependence online training.

As a result of our findings of overall poor quality of online courses, we suggest that course developers raise the quality and educational value of their courses by using the following strategies. First, subject matter experts must engage experts in instructional design to ensure course content is palatable for an online format and aligned with learning objectives. Second, interactive learning exercises need to be incorporated to maintain learner engagement and retention of course content [12]. Third, it is important to assess learners in a way that can facilitate knowledge transfer to real-world scenarios [12]. Simply providing learners with knowledge-based multiple-choice questions can be useful in particular educational settings; however, in CME courses it is crucial that application of knowledge is assessed. Finally, course designers need to remember that online courses require constant oversight and continuous improvement to keep course content and interactive learning activities current and fresh.

The potential to engage in meaningful reflection and optimize the multimedia potential to enhance cognitive, affective, and kinesthetic learning is the promise of e-learning. When developing an online course, it is important to remember that simply uploading text, charts, visuals, or a PowerPoint presentation to an online environment does not create an e-learning module. Unfortunately, this was the primary design approach for many of the courses reviewed. Modern education is characterized by the employment of multiple modalities to engage a wide variety of learners. Thus, effort needs to be made by course developers to utilize different learning approaches beyond didactic text, such as case simulations, quizzes, interactive learning objects, and discussion forums. Moreover, instructional design principles suggest that there needs to be alignment of the learning objectives, the content (including subcontent), the case scenarios, and assessments. Often, we observed misalignment of these essential elements—a design flaw risking disengagement of the learner from the material irrespective of the quality of the content. Additionally, pilot testing the e-learning module to evaluate both content and instructional design prior to implementation and widespread dissemination is imperative. Pilot testing the module in a sample of the target audience is an opportunity to garner valuable feedback and allow course developers to make improvements and enhance the module for future learners.

There are several limitations to our study. Although the rubric we chose was deemed to be the most thorough in assessing best practices and instructional design out of the 10 rubrics reviewed, there are limitations in its use. The rubric was designed for use in higher education online courses based on the assumption of a course management system that allows for learner interaction, such as learner-to-learner, learner-to-content, and learner-to-instructor interaction. Unfortunately, almost none of the courses we evaluated fostered such interaction. Although the rubric was designed to capture both design and content, there seemed to be a stronger focus on the e-learning environment and less on the content. This may prove to be a limitation as diverse delivery needs may be required for different content approaches. Both content and delivery are important to the outcomes, but the rubric assumes a higher weighting on delivery than content. In addition, replicating this evaluation may be challenging because the rubric requires trained, expert reviewers who have had years of experience working in tandem and cannot be generally applied for use by untrained reviewers. This also impacts the minimal changes scoring because the expert reviewer team relies on 15 years of experience in online course design and delivery to determine the minimal changes score. This score was determined by highlighting categories where minimal changes would provide maximum points. These subjective observations may prove to be problematic when attempting to quantify an overall numeric score. We also excluded from our review almost 40% (15/39) of the online courses we identified. Because these courses could not be accessed for meaningful review, we cannot comment on their quality or whether their inclusion in our study would have resulted in different conclusions. Finally, we cannot be certain that we identified all available English-language online learning courses because there is no comprehensive source for all courses on tobacco dependence treatment instruction. However, our search strategy mirrors that of most experienced online learners when seeking instructional content.

Our main conclusion is that there are important quality gaps between available online courses for tobacco dependence treatment instruction and the high quality that all courses should strive to achieve. Our evaluation makes it clear that there is a widespread lack of well-designed online continuing education courses in tobacco dependence treatment based on an analysis of instructional design quality. However, optimizing currently available online learning tools may not require extensive redesign or costly effort. Implementing modest changes will improve the quality of most existing courses to at least an average quality level and courses with average or above average quality may achieve superior-level quality with similar modest revision. The design elements that the majority of the courses lack are cohesiveness between the different module components and the linearity of the design. Because most courses in tobacco dependence treatment will be used by adult learners who are already working in a health care profession, course designers must also provide information succinctly and provide opportunity within the course for practice and problem solving. Simply providing abundant erudite content will not meet the needs of most adult learners. Having identified quality gaps in current online learning in tobacco dependence treatment instruction as well as feedback for developers of existing online courses, we would encourage course developers to employ these best practices and feedback for improvements to existing courses and in the design of new online learning opportunities. We believe there is a great unmet need for quality online education and that using instructional design principles could ensure greater impact of any content made available to online learners.

## Multimedia Appendix 1

Identified online tobacco cessation training programs.

## Multimedia Appendix 2

(Sample) Request for participation in the online course evaluation.

## Abbreviations

CME: Continuing Medical Education
