# Supplementary material for: Review and Evaluation of Online Tobacco Dependence Treatment Training Programs for Health Care Practitioners
Source: J Med Internet Res. 2015 Apr 17;17(4):e97. doi: 10.2196/jmir.3284 (PMC4417133; doi:10.2196/jmir.3284)
Supplement: Supplementary file 1 [file jmir_v17i4e97_app1.pdf]

Multimedia Appendix 1. Identified online tobacco cessation training programs

Course Components and URL	Audience	Developed/Offered by	CME	Cost
**Tobacco Free Futures: Brief Tobacco Intervention in Healthcare Settings**<br>Single Module: 1.5-2 hours<br>http://www.tobaccofreefutures.ca/online_training.html	Professionals working with tobacco users	Alberta Health Services, Canadian Cancer Society	None	Free
**The Brief Tobacco Intervention: Helping Alaskans Quit**<br>Single Module: 1 hour<br>www.akbriefintervention.org	Professionals working with tobacco users	Alere Wellbeing and American Academy of Family Physicians	Up to 1 CME/ CEU credit	Free
**Arkansas Addressing Tobacco Use with Your Patients**<br>Single Module: 1 hour<br>www.arkansasstop.org	Professionals working with tobacco users	Alere Wellbeing and American Academy of Family Physicians	Up to 1 CME/ CEU credit	Free
**Effecting Change with the 2As and R: The Brief Tobacco Intervention for Substance Abuse and Mental Healthcare Providers**<br>Single Module: 1 hour<br>www.dctobaccointervention.org	Professionals working with tobacco users	Alere Wellbeing and American Academy of Family Physicians	Up to 1 CME/ CEU credit	Free
**The 2As+R Brief Tobacco Intervention Training**<br>Single Module: 1 hour<br>www.helppatientsquitsc.org	Professionals working with tobacco users	Alere Wellbeing, South Carolina Department of Health, South Carolina Tobacco Quitline and Environmental Control and American Academy of Family Physicians	Up to 1 CME/ CEU credit	Free
**The 2A and R Brief Tobacco Intervention**<br>Single Module: 1 hour<br>www.2aandr.org	Professionals working with tobacco users	Alere Wellbeing, TCRC (Tobacco Cessation Resource Center) and American Academy of Family Physicians	Up to 1 CME/ CEU credit	Free
**The Washington State Tobacco Quit Line and Fax Referral Program**<br>Single Module: 1 hour<br>www.waquitlinetraining.org<br>www.faxreferral.org	Professionals working with tobacco users	Alere Wellbeing, TCRC (Tobacco Cessation Resource Center) and American Academy of Family Physicians	Up to 1 CME/ CEU credit	Free
**The University of Massachusetts Basic Skills for Working with Smokers Program**<br>Nine modules: no time frame available<br>http://www.umassmed.edu/tobacco/training/basicskills_online/p	Professionals working with tobacco users	American Dental Hygienists' Association	None	$150/person
**Freedom from Smoking Online Program**<br>Eight modules, 4 lessions/module: no time frame available<br>http://www.ffsonline.org/	Professionals working with young tobacco users	American Lung Association	None	Basic Program (free)

**CTIP**<br>6 modules: no time frame<br>www.tobaccoed.org/ctip/	Professionals working with tobacco users	BC Cancer Agency and TobaccoEd.org	None	Free
**Quit Using and Inhaling Tobacco (QUIT) Smoking Cessation Program**<br>5 modules: 5 hours<br>http://www.pharmacists.ca/index.cfm/education-practice-resources/professional-development/quit/quit-continuing-education/	Pharmacists providing smoking cessation services	Canadian Pharmacists Association	QUIT Online (5.0 CEUs), QUIT Live (6.0 CEUs)	CPhA member $75 plus taxes<br>Non-member $150 plus taxes
**Help your Clients from gambling with their Health – You can make a difference!**<br>Course is no longer accessible<br>http://www.cdha.ca/AM/Template.cfm?Section=Tobacco_Cessation&Template=/CM/HTMLDisplay.cfm&ContentID=10591	Dental professionals working with tobacco users	Canadian Dental Hygienists Association (CDHA)	None	CDHA member$100.0<br>CDHA student : $50.00<br>Non-member fee: $259.00
**Provincial Concurrent Capable Learning Series (PCCLS)**<br>4 modules over 13 weeks: 18 hours<br>http://www.addictioncentre.ca/?page_id=612	Professionals working with young tobacco users	Alberta Health Services	18 Mainpro-M1 credits	Undisclosed Fee
**Smoking Cessation for Pregnancy and Beyond: Learn Proven Strategies to Help Your Patients Quit, A Virtual Clinic**<br>Virtual learning clinic with 5 sections: 4 hours<br>https://www.smokingcessationandpregnancy.org/	Professionals working with female tobacco users	Dartmouth Medical School, American College of Obstetricians and Gynecologists, supported by CDC	Up to 6 hours of CME credit, up to 4 hours (4.5 CE credits).	$25.00
**Online Tobacco Training**<br><br>**LaraSig Training for Medical Students and Professionals (Florida AHEC Network)**<br>7 core modules and 6 optional modules: up to 8.25 hours<br>www.larasig.com<br><br>**Tobacco Training and Cessation Program**<br>3 core modules and 7 optional modules up to 10 hours<br>www.aheceducation.com	Professionals working with young tobacco users	Everglades Area Health Education Center (EAHEC)	CME Certificate (claim credit for amount of time spent on activity)	Free
**CME Online short courses**<br>14 modules: 1 hour each<br>http://www.aheceducation.com/ELearning/CourseCatalog.aspx	Professionals working with young tobacco users	Florida Keys AHEC	1 per course	Free
**AHEC Tobacco Training and Cessation (ATTAC)**<br>Number of modules not disclosed<br>http://www.gnahec.org/program-areas/tobacco-cessation/attac/	Professionals working with young tobacco users	GNAHEC (Gulfcoast North Area Health Education Center, Inc.)	None	Free
**Ask About the Elephant**.<br>1 module: 1 hour<br>http://learnonline.health.nz/login/index.php	Professionals working with tobacco users	Hayden McRobbie/Royal New Zealand College of General	Up to 1 hour	Free

		Practitioners		
**Global Tobacco Control**<br>11 modules: no time frame<br>http://globaltobaccocontrol.org/online_training	Professionals working with tobacco users	Johns Hopkins Bloomberg School of Public Health	3 credits	Free
**Health Communication Programs for Tobacco Control**<br>As part of the Global Tobacco Control certificate program, covers 5 topics<br>http://www.globaltobaccocontrolalumni.org/Certificate_Program/cp_curriculum.html	Professionals working with tobacco users	Johns Hopkins Bloomberg School of Public Health	3 credits	Free
**Tobacco Prevention, Cessation**<br>3 hours<br>http://cecourses.org/drug-abuse/tobacco-prevention/	Professionals working with tobacco users	LearnWell.org Continuing Education In Health and Ethics	3.00 CE Credit Hours (0.3 CEUs)	$24.00
**Helping your Pregnant Patients Stop Smoking.**<br>5 lectures, 4 case simulations: 2.4 hours<br>http://musom.marshall.edu/medctr/med/tobaccocessation/pregnancyandsmoking/login.aspx	Professionals working with female tobacco users	Marshall University School of Medicine	2 credits	Free
**NCSCT (UK) Training and Assessment Programme.**<br>No details provided regarding length or time<br>http://www.ncsct.co.uk/training/ncsct-training-faqs/ncsct-training-and-assessment-programme	Professionals working with tobacco users	National Centre for Smoking Cessation and Training - UK	None	Free
**The Brief Tobacco Intervention Training for Tobacco Smoking Project**<br>4 modules: at your own pace<br>http://ndri.curtin.edu.au/btitp/	Professionals working with tobacco users	National Drug Research Institute (Australia), Smoke Free WA Health	None	Free
**Counseling for Change: An Online Tobacco Cessation Course**<br>4 objectives: 5 hours<br>http://northwestahec.wfubmc.edu/mura/www/#/event/26290	Professionals working with tobacco users	Northwest AHEC	None	$20 fee to receive certificate)
**Tobacco Dependence and Treatment Online CEUs**<br>5 course modules: hours vary from 3-6 hours<br>http://www.quantumunitsed.com/online-ceus/tobacco-dependence-and-treatment.php		Quantum Units Education	3 CEU hours<br>5 CEU hours<br>5 CEU hours<br>3 CEU hours<br>6 CEU hours	$9.00<br>$15.00<br>$15.00<br>$9.00<br>$18.00
**Tobacco Intervention and the Healthcare Provider**<br>Course expired<br>http://www.texmed.org/template.aspx?id=8461	Primary care physicians working with tobacco users	Texas Medical Association, Cancer Prevention and research Institute of Texas	*1 AMA PRA Category 1* credit	Free
**Tobacco and Public Health: From Theory to Practice**<br>4 modules: 2 hours each<br>http://tobaccocourse.otru.org/	Busy professionals working with tobacco users	The Ontario Tobacco Research Unit	None	Free
**Tobacco Recovery Resource Exchange E-Learning – Integrating Tobacco Use Interventions into Chemical Dependence Services**<br>5 modules: ranging from 3-5 hours per module<br>http://www.tobaccorecovery.org/LMS/HUB/index.cfm	Professionals working with tobacco users	Tobacco Recovery Resource Exchange, Professional Development Program (Rockefeller College) University of Albany	None but receive OASAS Credits: 3 - 5 hours CASAC, CPP, CPS depending on module (7 modules)	Free, requires registration
**Performance Improvement Activity** – CS2day –	Professionals working	U of WI School of	Up to 20	Free

Cease Smoking Today<br>20 hours<br>https://www.cs2dayuwpi.com/default.aspx	with tobacco users	Medicine & Public Health and CS2Day – 9 partner orgs	credits	
**Intake, Assessment and Treatment Planning for Tobacco Cessation.**<br>Brief guide for conducting an intake assessment: 45 minuters<br>http://www.umassmed.edu/tobacco/training/intake.aspx	Primary care physicians, nurse practitioners, physician's assistants and registered nurses working with tobacco users	UMass Center for Tobacco Treatment Research and Training	None	Free
**Basic Skills for Working with Smokers**<br>9 modules: self-paced online course<br>http://www.umassmed.edu/tobacco/training/basicskills_online.aspx	Professionals working with tobacco users	UMass Medical School	CEU's available	$150
**Tobacco Cessation Online Learning Center**<br>Course is no longer accessible<br>http://banduraold.sbs.arizona.edu/hcp/olc/main/Orientation1.html	Professionals working with tobacco users	University of Arizona	None	Free with Virtual Library Card
**Rx for Change: Clinician-Assisted Tobacco Cessation**<br>Complete program requires 12 hours<br>http://rxforchange.ucsf.edu/about.php	Training students and licensed clinicians working with tobacco users	University of California, San Francisco	None	Free
**Smoking Cessation Leadership Center (SCLC) Webinars.**<br>24 webinars: 90 minutes each<br>http://smokingcessationleadership.ucsf.edu/Webinars.htm	Professionals working with tobacco users	University of California San Francisco	1.5 per course	$25.00 (free if you do not want CME?)
**Tobacco Use and Dependence: A 2011 Update of Treatments**<br>1 hour online module<br>http://www.medscape.org/viewarticle/757167	Professionals working with tobacco users	UW – CTRI, developed and funded by Medscape	Physicians: 1 *AMA PRA*<br>Nurses: 1 *ANCC*<br>Pharmacists: 1 *ACPE (0.1 CEUs)*	Free
**Rx for Change: Clinician-Assisted Tobacco Cessation program for patients with cancer.**<br>2 hour online program<br>https://tobacco.ucsf.edu/content/rx-change-clinician-assisted-tobacco-cessation	Professionals working with tobacco users	Walther Cancer Institute Foundation	CE/CME offered to pilot study participants	Free
**Quit Tobacco Make Everyone Proud**<br>Online support program for US military service members and Veterans.<br>http://www.ucanquit2.org/	Tobacco users	US Department of Defense	None	Free
**YourChoice Online Tobacco Course**<br>Self-paced learning module<br>http://www.manateeyourchoice.com/online-tobacco.html	Tobacco users	Your Choice Manatee Health Plan	None	$25 registration cost
