# Supplementary material for: Review and Evaluation of Online Tobacco Dependence Treatment Training Programs for Health Care Practitioners
Source: J Med Internet Res. 2015 Apr 17;17(4):e97. doi: 10.2196/jmir.3284 (PMC4417133; doi:10.2196/jmir.3284)
Supplement: Supplementary file 2 [file jmir_v17i4e97_app2.pdf]

Multimedia Appendix 2. (Sample) Request for participation in the online course evaluation

November 1, 2012

To: Permissions Department<br>[Address]	From: Karina Goncharenko<br>karina.goncharenko@camh.ca<br>Research Analyst, TEACH Project, NDC<br>Centre for Addiction and Mental Health<br>175 College Street,<br>Toronto, ON M5T1P7<br>Tel: (416) 535-8501 x. 7437

Dear Editor/Course Administrator,

The Global Bridges Project (an international collaboration between the Mayo Clinic and the Centre for Addiction and Mental Health), is focused on increasing capacity for tobacco dependence treatment services. We are conducting a comprehensive research review of existing online, English-language tobacco dependence treatment courses targeting health and allied health practitioners, and are seeking your participation. Your course is among 39 online courses that we have identified.

No prior review has been done, to our knowledge, of the extent to which existing online courses incorporate best practices in online teaching/learning and instructional design. Assessments will be carried out by an independent evaluation team at Towson University, from November 2012-January 2013. The evaluation team will be using a well-validated and established rubric, “Peer Review Rubric for Online Learning”. This rubric is based on documented quality indicators for online courses, and has been used in other research studies assessing excellence in instructional design and best practices.

The assessments will sample all available online courses who agree to participate, and only aggregate results will be published without specific course identifiers. That is, the names and links for all participating and non-participating courses will be published without any identifying information relating to individual course evaluation outcomes.

If you decide to participate in this study, you will have the option of receiving confidential feedback/evaluation ratings and commentary about the evaluation outcomes relating to **your** course. In addition, we will provide you with information and contact resources about how to elevate and enhance the quality of your course, should you wish to do so. There will be no costs to your organization for this information.

If you decide to participate and your course has a registration or enrolment fee, we ask that you waive this fee and/ or provide access to your materials for the purposes of the evaluation. Your course content or associated materials will not be shared externally by the independent evaluation team. If you do not own the copyright for the online course materials about which we are inquiring, we would appreciate any information that you can provide concerning the proper person or organization that we can contact.

If you decide not to participate you will be included in the “declined participation” group which will be shared in the publication.

Thank you for your time and consideration of this request. We look forward to hearing from you at your earliest convenience.

Sincerely,

TEACH Project, CAMH
